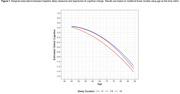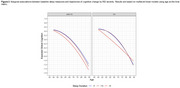# Sleep duration predicts 14‐year cognitive decline: A preliminary analysis of the Hispanic Community Health Study/Study of Latinos

**DOI:** 10.1002/alz70860_105332

**Published:** 2025-12-23

**Authors:** Alberto R Ramos, Wassim Tarraf, Charles Decarli, Martha L Daviglus, Galit L Dunietz, Linda C Gallo, Kevin A Gonzalez, Youngmee R Kim, Yasmin Mossavar‐Rahmani, James R Pike, Susan Redline, Daniela Sotres‐Alvarez, Douglas M Wallace, Hector M Gonzalez

**Affiliations:** ^1^ University of Miami Miller School of Medicine, Miami, FL, USA; ^2^ Wayne State University, Detroit, MI, USA; ^3^ University of California, Davis, Davis, CA, USA; ^4^ IDeA Laboratory, Department of Neurology, UC Davis, Davis, CA, USA; ^5^ University of Illinois at Chicago, Chicago, IL, USA; ^6^ University of Michigan, Ann Arbor, MI, USA; ^7^ San Diego State University, San Diego, CA, USA; ^8^ University of California, San Diego, La Jolla, CA, USA; ^9^ University of Miami, Miller School of Medicine, Miami, FL, USA; ^10^ Albert Einstein College of Medicine, Bronx, NY, USA; ^11^ University of North Carolina, Chapel Hill, NC, USA; ^12^ Brigham and Women's Hospital, Harvard Medical School, Boston, MA, USA; ^13^ University of Miami, miami, FL, USA

## Abstract

**Background:**

Sleep is important for brain health in aging adults and offers opportunities to prevent or treat cognitive decline. Our published research from the Hispanic Community Health Study/Study of Latinos (HCHS/SOL) showed associations between sleep duration, particularly long sleep, and neurocognitive decline over seven years. It is unknown whether these relationships persist over time or are influenced by sleep apnea severity, which can lead to either short or long sleep durations. We aimed to examine the association between baseline sleep duration and cognitive trajectories over time (14years of follow‐up) and to determine whether these associations varied by sleep apnea severity.

**Methods:**

In this preliminary analysis of SOL‐INCA participants, a cognitive ancillary study to HCHS/SOL, we analyzed the associations of baseline sleep measures from HCHS/SOL (sleep questionnaires: *n* = 2,521; home sleep apnea testing: *n* = 2,389) with baseline and follow‐up cognitive assessments made by the SOL INCA ancillary studies (2015–2018 and 2022 and 2024). We use multilevel linear models, with age as the time metric, to test how baseline sleep duration (<7, 7‐9, and 9+ hours) associated with trajectories of cognitive change over 14years (on average) and whether these associations differed by baseline sleep apnea severity, based on the respiratory event index 3% saturation (REI <15 vs. 15+). Models controlled for sex, Hispanic/Latino heritage, and apolipoprotein, APOE4 (any 4 vs. none).

**Results:**

The sample age: 54.4±SD (mean unweighted). The unweighted mean sleep duration for the sample was 7.8 (range: 3 ‐ 13.5) hours. The unweighted mean for REIwas7.5 (range: 0 ‐ 116.5) A sequential comparison of random intercepts and slopes revealed a significant association between sleep duration and quadratic patterns of cognitive decline. Individuals reporting 9+ hours of sleep (vs. < 9 hours) at baseline had a faster cognitive decline over time (Figure 1). The associations were not statistically differentiated by sleep apnea severity (Figure 2).

**Conclusion:**

These preliminary findings how that long sleep duration at baseline is associated with greater cognitive decline over 14 years. This association does not vary with sleep apnea severity. These results highlight the need to promote healthy sleep duration to support brain health and reduce cognitive decline.